# Blood Lead Levels and Learning Disabilities: A Cross-Sectional Study of the 2003–2004 National Health and Nutrition Examination Survey (NHANES)

**DOI:** 10.3390/ijerph14101202

**Published:** 2017-10-10

**Authors:** David A. Geier, Janet K. Kern, Mark R. Geier

**Affiliations:** 1 Institute of Chronic Illnesses, Inc., 14 Redgate Ct, Silver Spring, MD 20905, USA; davidallengeier@comcast.net (D.A.G.); mgeier@comcast.net (M.R.G.); 2 CoMeD, Inc., Silver Spring, MD 20905, USA; 3 CONEM US Autism Research Group, Allen, TX 75013, USA

**Keywords:** lead, learning disability, neurodevelopmental disorder, NHANES

## Abstract

Difficulties in the acquisition and use of listening, speaking, reading, writing, reasoning or mathematical abilities are present among persons diagnosed with learning disabilities (LDs). Previous studies suggest a significant relationship between lead (Pb) exposure and LDs. This study evaluated the potential dose-response relationship between blood Pb levels and the risk of LDs. This cross-sectional study examined 1411 children (32,788,743 weighted-persons) between 6 and 15 years old from the 2003–2004 National Health and Nutrition Examination Survey (NHANES) by analyzing demographics, health related-questions, and laboratory tests using survey logistic and frequency modeling in SAS. On a µg Pb/dL basis, a significant dose-dependent relationship between increasing blood Pb levels and increasing risk of LDs was observed (odds ratio (OR) = 1.21, 95% confidence interval (CI) = 1.03–1.43). The relationship remained significant when examining covariates such as gender and race (OR = 1.19, 95% CI = 1.00–1.40). By contrast, no dose-dependence was observed between increasing blood Pb levels and the risk of hay fever in the last year (OR = 0.77, 95% CI = 0.56–1.056), a non-plausibly biologically related outcome of blood Pb levels. Persons in the 50th–75th (12.80%) and 75th–100th (17.14%) percentiles of blood Pb were significantly more likely to have LDs than persons in the 0–50th percentile of blood Pb (8.78%). An estimated 1 million persons born in the US from 1989 to 1998 developed LDs from elevated blood Pb levels. Overall, this study revealed a significant dose-dependent association between increasing childhood blood Pb levels and the risk of a LD diagnosis, but it was not possible to ascribe a direct cause-effect relationship between blood Pb exposure and LD diagnosis. Childhood Pb exposure should be considered when evaluating children with LDs, and continuing efforts should be made to reduce Pb exposure.

## 1. Introduction

Among individuals diagnosed with learning disabilities (LDs), there are difficulties in the acquisition and use of listening, speaking, reading, writing, reasoning, or mathematical abilities. According to the United States (US) Agency for Toxic Substances and Disease Registry (ATSDR), low doses of lead (Pb) can cause a broad range of functional problems, including many different types of LDs [1]. The ATSDR also described that many children diagnosed with LDs as a consequence of Pb exposure perform poorly in school [1]. Previous studies suggest a significant relationship between Pb exposure and LDs [1,2]. For example, Kaiser et al. [3] used extant datasets from the Florida Department of Health, Childhood Lead Poisoning Prevention Program and the Florida Department of Education to compare children who were tested for Pb poisoning to children not tested for Pb poisoning. They used special education categories as a proxy for developmental disabilities. Children screened for Pb poisoning were more likely to be receiving services for behavior problems, mental retardation, LDs, and speech-language impairment than other children attending the same schools. In addition, studies examining blood Pb levels reveal a significant relationship between increased blood Pb levels and increasing risk of LDs [4].

Although steps have been taken to reduce Pb exposure in the past half century, and estimates of children and pregnant women with high blood Pb levels have dropped significantly in recent decades, it still remains a public health problem [1,5,6]. Exposure to Pb still occurs, and about 250,000 children in the US have blood Pb levels over the current Level of Concern of 10 µg Pb/dL [6]. Moreover, a recent review of the scientific literature on Pb by Vorvolakos et al. [7] found that there is no safe threshold for Pb exposure. Other studies and the ATSDR confirm this finding [1,2]. Landrigan et al. [8] estimated the contribution of environmental pollutants to the incidence, prevalence, mortality, and costs of pediatric disease in American children and estimated the total annual costs for Pb poisoning to be $43.4 billion.

In this study, the National Health and Nutrition Examination Survey (NHANES) was examined. The NHANES is a program of studies designed to assess the health and nutritional status of adults and children in the US. The survey is unique in that it combines interviews with physical and laboratory examinations. NHANES is a major program of the National Center for Health Statistics (NCHS) of the US Centers for Disease Control and Prevention (CDC). The NHANES program began in the early 1960s, and has been conducted as a series of surveys focusing on different population groups or health topics. In 1999, the survey became a continuous program with components that are adaptable to a variety of health and nutritional measurements in order to meet emerging needs. The NHANES examines a nationally representative sample of about 5000 Americans each year, and these persons are located in counties across the US, 15 of which are visited each year. As described by NCHS, and consistent with the objective of this study, NHANES is supposed to be used in epidemiological studies to determine the prevalence of major diseases and risk factors for diseases, which help develop sound public health policy.

The current study examined the 2003–2004 NHANES data to evaluate the potential relationship between blood Pb levels and the risk of LDs. The 2003–2004 NHANES data was analyzed because it was the most recent NHANES data collected in which blood Pb levels and LD status were recorded. It was hypothesized that increasing blood Pb levels would be significantly associated with LDs in a dose-dependent fashion. Then, based upon the results observed, extrapolations were made to determine the overall number of persons with LD diagnoses in the US as a consequence of elevated blood Pb levels.

This study is different from previous studies in a number of aspects. First, it not only examined the risk of elevated blood Pb levels, but evaluated whether the relationship was dose-dependent. Second, it also provided important insights into the number of LD diagnoses associated with elevated blood Pb levels among the general US childhood population. Finally, this study examined a number of covariates to determine, what, if any impact they may have had on the relationship between elevated blood Pb levels and the risk of LDs.

## 2. Methods

This study integrated NHANES data that included demographic, health related-questions, and laboratory tests.

### 2.1. Study Participants

At total of 10,122 persons were examined from the 2003–2004 NHANES. Among this overall population, a sub-population was identified of 1411 children (between 6 and 15 years-old) with non-missing gender, age, race, diagnosis status for LDs, and blood heavy metal test results for Pb. Then, to this subset of persons, the full sample 2 years interviewed and medical examined weight variable (variable: WTMEC2YR) was applied to the 2003–2004 NHANES data. This yielded a total of 32,788,743 weighted-persons examined in the current study.

In NHANES, a sample weight is assigned to each sample participant. The sample weight is a measure of the number of persons in the target population that the sampled individual represents. Sample weights are needed to obtain unbiased estimates of population parameters when the sample participants are chosen with unequal probabilities.

The demographics NHANES dataset was examined for gender (variable: RIAGENDR (male = 1, female = 2)), age in years at screening (variable: RIDAGEYR), and race (variable: RIDRETH2 (non-Hispanic White, non-Hispanic Black, and Hispanic = Mexican American + Other Hispanic)).

### 2.2. Outcomes

The medical conditions NHANES dataset was examined for diagnosis status of LDs (variable: MCQ060 [Yes = 1, No = 2], survey question, “Has a representative from a school or a health professional ever told {you/SP} that {s/he/SP} had a learning disability”). In addition, the medical conditions NHANES dataset was examined for the diagnosis status of hay fever within the last year (variable: MCQ120A [Yes = 1, No = 2], survey question, “During the past 12 months, {have you/has SP} had…hay fever”). This outcome was selected *a priori* as an outcome not plausibly biologically related to blood Pb levels.

### 2.3. Exposures

The laboratory NHANES dataset was examined for blood Pb (variable: LBXBPB, limit of detection = 0.2 µg Pb/dL). Values less than the detection limit were assigned a value of 0.2 µg Pb/dL. Whole blood Pb concentrations were determined using inductively coupled plasma-mass spectrometry (ICP-MS). This multi-element analytical technique is based on quadrupole ICP-MS technology. In this method of analyzing whole blood Pb concentrations, blood samples were diluted with 18 Mega-ohm water and with diluent, which contained 1% *v/v* tetramethylammonium hydroxide (TMAH), 0.5% disodium ethylenediamine tetraacetate (EDTA), 10% ethyl alcohol, 0.05% Triton X-100. Bismuth (Bi) was added for internal standardization of Pb. The samples were prepared with the following ratio: Sample:Water:Diluent = 1:1:48 correspondingly.

### 2.4. Statistical Analyses

This study employed the SAS system for Windows, version 9.4 (Cary, NC, USA), running on an 64-bit PC with dual core Intel^®^ (Santa Clara, CA, USA) Xeon^®^ CPU ×5680 at 3.33 GHz, 6 cores, and 12 logical processors, with 44.0 GB of RAM, and utilizing Microsoft (Redmond, WA, USA) Windows 7 Ultimate operating system to examine the 2003–2004 NHANES data.

In all statistical analyses, the statistical package in SAS was utilized, and a two-sided *p*-value < 0.05 was considered statistically significant. The persons examined in this study were evaluated to determine the potential relationship between blood Pb levels and the risk of LDs or hay fever in the last 12 months. The null hypothesis was that there would be no relationship between blood Pb levels and the risk of LDs or hay fever within the last year.

Initially, using survey logistic regression modeling (stratum-variable: SDMVSTRA, cluster-variable: SDMVPSU, and weight-variable: WTMEC2YR) in SAS, the potential relationship between blood Pb levels and the risk of LDs was determined for the persons examined (Model I). Then a SAS survey logistic regression model (stratum-variable: SDMVSTRA, cluster-variable: SDMVPSU, and weight-variable: WTMEC2YR) was developed to include, in addition to blood Pb levels, other standard covariates such as gender and race, to determine, what, if any, effect these covariates had on the potential relationship between blood Pb levels and the risk of LDs (Model II). This later model (Model II) was also employed to evaluate the potential relationship between blood Pb levels and the risk of hay fever in the last year.

Subsequently, the data were analyzed on a percentile basis for blood Pb levels for the risk of LDs. The data were separated using SAS with the 0 to 50th percentile blood levels as the reference exposure group, and with the increasingly exposed groups being those with blood levels in the 50th to 75th percentile group and the 75th to 100th percentile group. The data were then analyzed in SAS by survey frequency modeling (stratum-variable: SDMVSTRA, cluster-variable: SDMVPSU, and weight-variable: WTMEC2YR). The Rao-Scott χ^2^ test statistic was employed to determine statistical significance.

## 3. Results

Table 1 displays the overall composition of the 1411 children (weighted n = 32,788,743) examined in this study. There were slightly more males than females (male/female ratio = 1.10) with a mean age of 10.71 years-old and a mean birth year of 1993 among the children examined. The children examined were racially composed of (in descending order of frequency): non-Hispanic Whites (62.28%) > Hispanics (19.60%) > non-Hispanic blacks (18.12%). It was observed among the children examined that 11.93% (or about 1 in 8.4 persons) had a LD and 12.05% (or about 1 in 8.3 persons) had hay fever in the last year.

Table 2 reveals the relationship between blood Pb levels and the risk of LDs using survey logistic regression models. It was observed in all models (Models I and II) that increasing blood Pb levels were a significant dose-dependent risk factor for LDs. Among the various covariates studied, it was found that gender (being a male) was associated with an increased risk for LDs in constructed survey logistic regression Model II. No significant relationships were observed for race covariates and the risk of LDs in survey logistic regression Model II. Finally, it was observed using Model II that there was no dose-dependent relationship between blood Pb levels and the risk of hay fever in the last year (odds ratio = 0.77, 95% confidence interval = 0.56 to 1.06, *p* = 0.10).

Table 3 displays the prevalence ratios for LDs observed for increasing percentiles of blood Pb levels in comparison to a 0 to 50th percentile reference group. It was determined that the prevalence ratio = 1.46 in the 50th to 75th percentile group and the prevalence ratio = 1.95 in the 75th to 100th percentile group were significantly increased in comparison to the reference 0 to 50th percentile group.

## 4. Discussion

The results observed in this study revealed a dose-response relationship between increasing blood Pb levels and an increasing risk of LDs. This relationship was confirmed using survey logistic regression and survey frequency modeling. Furthermore, it was observed that when considering covariates, such as gender and race, the relationship between increasing blood Pb levels and increasing risk of LDs remained significant. Finally, when considering the outcome of hay fever in the last year, *a priori* selected to not be plausibly biologically related to blood Pb levels, no significant dose-dependent relationship was observed.

The results observed in the current study are not only consistent with the aforementioned studies [1,2,3,4,9], but they provide additional important insights into the potential attributable population consequences of elevated blood Pb levels among the general US childhood population. The results of the present study revealed that children in the 50th to 100th percentile of blood Pb levels (1.007 µg Pb/dL to 13.50 µg Pb/dL), when compared with children in the 0 to 50th percentile of blood Pb levels (0.2 µg Pb/dL to 1.007 µg Pb/dL), were significantly more likely to have a LD (prevalence ratio = 1.71, 95% confidence interval = 1.18 to 2.49, *p* = 0.0007, prevalence attributable rate = 0.062). Therefore, it was possible to estimate from the number of weighted-persons examined that 1,025,695 persons born from 1989 to 1998 were diagnosed with LDs attributable to increased blood Pb levels in the US.

Also, since a total of 3,911,498 weighted-persons with LDs were examined, and 1,025,695 weighted persons with LDs were attributable to increased blood Pb, this means that 26.2% of all LDs (or about 1 in 3.8 LDs) were associated with increased blood Pb levels. As a result, the majority of LDs are caused by other environmental and/or genetic factors [10,11]. This finding is plausible because there are other known toxicants that are associated with LDs. For example, it was estimated from a longitudinal cohort study examining medical records that during the decade 1991–2001, 0.5–1 million additional US children were diagnosed with specific delays in development as a consequence of exposure to organic mercury within the first 6 months of life [11].

In further considering the results observed in the current study, it is important to realize that they are biologically plausible because Pb, a known neurotoxin, has multiple detrimental effects on the brain. Pb undermines brain growth and function [12]. It disrupts multiple synaptic processes with long-term consequences for synaptic function and neuronal development [13]. Studies using animal models show that Pb induces detrimental changes in hippocampal *N*-methyl-d-aspartate receptor (NMDAR) subunit mRNA expression. In fact, the NMDAR, which is critical in learning and memory, is a principal target for Pb-induced neurotoxicity [14,15]. Further, Pb causes brain tissue damage through increased oxidative stress, inflammation, and apoptosis in the brain [12,16,17].

Because Pb causes brain tissue damage through increased oxidative stress, inflammation, and apoptosis in the brain, it makes sense that this study found that there were more males than females with LDs. Studies show that females are better able to shut down excessive brain inflammation and cell death [18]. Other studies have observed a greater effect of Pb exposure in boys. For example, Jedrychowski et al. [19] measured the accumulated Pb dose in infants over the pregnancy period using cord blood Pb level (BLL). They also used the Bayley Mental Development Index (MDI) to assess cognitive deficits. The results of the study suggested that there is no threshold for Pb toxicity in children, and that 3-year old boys were more susceptible to prenatal very-low-Pb toxicity than girls.

Unfortunately, childhood Pb exposures are also associated with long-term ramifications. Evidence suggests that childhood Pb exposure is associated with impaired cognitive function later in life [20].

## 5. Strengths/Limitations

A strength of this study was that the prevalence rate of LDs among children examined from the NHANES program in this study revealed consistency with other general US population assessments. For example, consistent with 12% of children having a LD in this study, previous investigators examining 3 to 17 year-olds in the US National Health Interview Survey (NHIS) for children born in years roughly corresponding to those examined in this study reported that about 8% of children had a LD [21].

An additional strength of this study was the special emphasis placed on ensuring the age of the persons examined were sufficiently old enough to guarantee that most persons with a LD diagnosis were captured. In this study, children examined were between 6 and 15 years old. The lower age of the persons examined was selected based upon a previous study revealing that the mean age of initial diagnosis of LD was 2.62 years-old, and the standard deviation of mean age of initial diagnosis was 1.58 [22]. It was assumed that, in a population with a Gaussian distribution for the mean age of initial LDs diagnosis, persons at least 6 years old would be at less than a 2.5% chance of being diagnosed with a LD with increasing age (i.e., the aforementioned mean age of initial diagnosis + 2 × standard deviation of the mean age of initial diagnosis). As a consequence of the minimum age of children examined in this study being 6 years old, there was a minimal chance, based upon their age, that a LD diagnosis had yet to be made.

Another strength of this study was that the NHANES data was collected independently of the methods used to analyze the data in this study. Further, the different NHANES data elements were also collected independently from one another (i.e., persons providing information about their LD diagnosis status were not aware of their blood Pb levels, and those measuring blood Pb levels were not aware of the LD diagnosis status of the samples tested). As a result, phenomena such as selection bias for study participants or recall bias or examiner bias should have minimally impacted the phenomena observed in this study.

A further strength of this study was the consistency and specificity of the results observed. It was consistently observed in this study that increasing blood Pb levels were associated with an increased risk of a LD diagnosis. This phenomenon remained consistent when using different methods of data analysis, or when statistical models with increasingly complex covariates were employed. The specificity of the phenomenon observed in this study was further confirmed when the statistical models developed were used to evaluate the increasing risk of the outcome of hay fever within the last year with increasing blood Pb levels. It was determined a priori that increasing risk of hay fever within the last year should not be plausibly biologically linked to increasing blood Pb levels. The same survey logistic regression modeling with the covariates of race and gender that revealed a significant relationship between increase in risk of LDs and increasing blood Pb levels failed to find any relationship between increased risk of hay fever within the last year and increased blood Pb levels.

A potential limitation of this study was that it was not possible to ascribe a direct cause and effect relationship between blood Pb exposure and a LD diagnosis. This potential limitation was a consequence of the fact that the NHANES data was collected as a cross-sectional study. Therefore, it was not possible to temporally follow the children examined over time to evaluate the precise timing between exposure and diagnosis. Despite this limitation, the results observed in this study are biologically plausible, and are supported by previous studies linking increased blood Pb levels with a LD diagnosis. Furthermore, a recent study by Gump et al. [9] revealed, consistent with the results observed in the present study, a significant relationship between increasing blood Pb levels and LD-associated psychological and behaviors among children of similar age (9 to 11 years-old) and similar blood Pb levels (0.19 to 3.25 µg Pb/dL) to those examined in this study. It is recommended that future studies further explore the phenomena observed in this study with other populations.

Another potential limitation of this study was the data collection methods utilized in the NHANES data examined. The outcomes examined in this study were based upon detailed survey questions asked of persons participating in the NHANES program. It is possible that those participating in the NHANES program may have recalled information erroneously (e.g., the diagnosis of LD) or reported information inaccurately (e.g., race). It is also possible that the laboratory tests conducted in the NHANES program may have yielded spurious results. Despite such limitations, it is presumed that such limitations/errors in the data examined would have applied equally to all the persons examined in this study. If anything, such errors in the data examined would have, in all probability, reduced the overall statistical power of this study to detect true significant relationships.

A still further limitation of this study was that only the 2003–2004 NHANES data were analyzed, and that different years of examination of persons may be important to the phenomena observed in this study. This year of NHANES data was analyzed because 2003–2004 was the only year in which there was consistency in the testing methods used to measure blood Pb levels, LD diagnosis status, and the persons tested were sufficiently old enough (i.e., at least 6 years-old). It would be worthwhile, in future studies, to not only examine Pb levels among persons with LDs in comparison to those without, but also to see how such levels fluctuate depending upon the data collection year.

An additional potential limitation of this study was that blood Pb levels were assumed to reflect ongoing environmental exposure to Pb. It is possible that there may be differences based upon the diagnostic status of the persons examined that may influence their blood Pb levels independent of random environmental exposure. For example, persons diagnosed with LDs may have impaired Pb detoxification pathways in comparison to persons without a LD diagnosis. As a consequence, this may significantly impact their blood Pb levels in comparison to those without a LD diagnosis. It would be worthwhile, in future studies, to further explore these phenomena.

Finally, there may be other factors that interact with blood Pb levels and, as a consequence, may at least partially explain the association observed between increasing blood Pb levels and the increasing risk of a LD diagnosis. For example, certain racial groups may be more likely to live in poor communities with increased environmental Pb exposure, whereas other racial groups may be more likely to live in more wealthy communities with decreased environmental Pb exposure [23]. As a consequence, it is of importance that increasing blood Pb levels and increasing risk of a LD diagnosis remained consistent in the unadjusted (Model I) and the adjusted (Model II, with covariates for gender and race) models utilized in this study. Similarly, it is of importance that the use of the survey functions in SAS (considering the NHANES variables of stratum-variable: SDMVSTRA, cluster-variable: SDMVPSU, and weight-variable: WTMEC2YR) help to consider the potential impact of other factors such as geography, income, and age may have had on the data examined in this study. Furthermore, it is of importance that the relationship between increasing blood Pb and the increasing risk of a LD was dose-dependent. All of these considerations, support that the phenomena observed in this study are genuine and not the result of anomalous factors. It is recommended that future studies be conducted in other datasets with other models to observe if they yield consistent results with those observed in this study.

## 6. Conclusions

This cross-sectional study observed compelling new evidence to confirm and extend previous epidemiological studies finding a significant dose-dependent relationship between increasing blood Pb levels and the increasing risk of a LD diagnosis, but it is not possible, from the data examined in this study, to ascribe a direct cause and effect relationship between blood Pb exposure and a LD diagnosis. This study also estimated the number of persons diagnosed with a LD as a result of elevated blood Pb exposure. It was estimated during the approximately one decade of US birth cohorts examined in this study from 1989 to 1998 that about 1 million persons may have been diagnosed with a LD as a consequence of elevated blood Pb levels in the US. These numbers are all the more unfortunate, because the use of Pb is completely avoidable.

In the US, over the last several decades, substantial steps have been taken to significantly reduce exposure to Pb by eliminating it from gasoline, paint, and toys. It is recommended that every effort be undertaken to eliminate all childhood Pb exposure as soon as possible.

## Figures and Tables

**Table 1:** A summary of the composition of weighted persons (n = 32,788,743) examined in this study.

Parameter Examined	Measurement
Gender (%)	
Males	17,205,646 (52.47%)
Females	15,583,097 (47.53%)
Age/Birth Year	
Mean Age ± std (range = 6–15 years-old)	10.71 ± 2.84
Mean Birth Year ± std (1989–1998)	1993 ± 2.84
Race (%)	
Non-Hispanic White	20,419,410 (62.28%)
Non-Hispanic Black	5,942,135 (18.12%)
Hispanic	6,427,198 (19.60%)
Diagnosis Status (%)	
Diagnosed with Learning Disability	3,911,498 (11.93%)
Diagnosed with Hay Fever in the Last Year	3,952,024 (12.05%)
Mean Blood Heavy Metal Level ± std	
Pb (µg/dL)	1.32 ± 0.95

dL = deciliter, Pb = lead, µg = microgram, std = standard deviation of the mean.

**Table 2:** A summary of survey logistic regression models ^1^ generated in this study to examine the potential relationship between blood Pb levels and the risk of learning disabilities.

Model	Variable	Odds Ratio	95% Confidence Interval	*p*-Values
I	Blood Pb (µg/dL)	1.21	1.03 to 1.43	0.018
II	Blood Pb (µg/dL)	1.19	1.00 to 1.40	0.044
	Gender (female = 2 vs. male = 1)	0.45	0.246 to 0.83	0.011
	Race (non-Hispanic Black vs. non-Hispanic White)	1.01	0.65 to 1.56	0.59
	Race (Hispanic vs. non-Hispanic White)	0.83	0.54 to 1.27	0.30

^1^ The models employed used stratum, cluster, and weight. This table reveals that increasing blood Pb levels significantly increase the risk of learning disabilities, even when considering covariates. dL = deciliter, Pb = lead, µg = microgram.

**Table 3:** A summary of survey frequency modeling ^1^ to evaluate the potential relationship between increasing blood Pb levels and the risk of learning disabilities.

Blood Pb Level of Exposure (µg/dL)	Weighted Number Diagnosed with LD (%)	Weighted Total Number of Individuals	Outcome Measurement
0 to 50th Percentile [reference] (0.2 to 1.007)	1,426,322 (8.78)	16,245,275	
50th to 75th Percentile (1.007 to 1.53)	1,032,781 (12.80)	8,069,241	
Prevalence Ratio (95% CI)			1.46 (1.11 to 1.92)
Rao-Scott χ^2^			9.81
DF			1
*p*-value			0.0017
Prevalence Attributable Rate			0.040
75th to 100th Percentile (1.530 to 13.50)	1,452,394 (17.14)	8,474,227	
Prevalence Ratio (95% CI)			1.95 (1.16 to 3.29)
Rao-Scott χ^2^			8.63
DF			1
*p*-value			0.0033
Prevalence Attributable Rate			0.084

^1^ The models employed used stratum, cluster, and weight. This table shows that blood Pb levels in the 50th to 75th percentile group and the 75th to 100th percentile group were associated with a significantly increased risk of an learning disability diagnosis in comparison to the 0 to 50th percentile reference group. DF = degrees of freedom, dL = deciliter, LD = learning disabilities, Pb = lead, µg = microgram.
